# Serotonin Syndrome with Atypical Hypernatremia

**DOI:** 10.7759/cureus.3616

**Published:** 2018-11-19

**Authors:** Ashley N Rubin, Eduardo D Espiridion, Mohamad Kattan, Elizabeth C Desmarais

**Affiliations:** 1 Family Medicine, West Virginia School of Osteopathic Medicine, Lewisburg, USA; 2 Psychiatry, Frederick Memorial Hospital, Clear Spring, USA; 3 Surgery, West Virginia School of Osteopathic Medicine, Lewisburg, USA; 4 Internal Medicine, West Virginia School of Osteopathic Medicine, Lewisburg, USA

**Keywords:** serotonin syndrome, depression, selective serotonin reuptake inhibitors (ssri), hunter criteria, altered mental status, hypernatremia, hyponatremia

## Abstract

The incidence of serotonin syndrome in the United States is rising yearly. Providers should be aware of the useful diagnostic criteria and have a low threshold for utilizing such criteria to prevent increased morbidity and mortality. In this case, a 64-year-old female with a complex past medical history presented to the emergency department with an altered mental status after being found poorly responsive by her husband. Many of her symptoms aligned with the commonly used criteria for serotonin syndrome; yet, her complex past medical history and uncommonly elevated sodium levels veered her providers from arriving at this diagnosis earlier. This unique case illustrates that despite having specific criteria for diagnosis, serotonin syndrome can be a complicated diagnosis to make, particularly in the setting of multiple co-morbidities and polypharmacy. In addition, it is important to keep medication toxicities high on a differential diagnosis list and be aware of the criteria used for diagnosis. One of the easiest and most cost-effective methods to diagnosis is to start with a thorough history, physical exam, and medication reconciliation to address easily preventable medication adverse effects.

## Introduction

Serotonin syndrome is a feared life-threatening toxicity reaction due to increased serotonergic overload in the central nervous system. Serotonin (5-hydroxytryptamine or 5-HT) is a chemical neurotransmitter linked to overall happiness and well-being [1]. It is commonly found in the central nervous system, gastrointestinal tract, and platelets. Along with many notable functions in the body relating to mood and cognition, serotonin is a precursor to melanin and has been identified for its role in the sleep-wake cycle as part of the internal circadian rhythm system [1]. In the brain, serotonin impacts levels of mood, anxiety, and happiness [1]. The most well-studied aspect of serotonin’s effect on mood is the role it plays in depression [2]. It is unclear whether depression causes low levels of serotonin or whether low levels of this neurotransmitter induces depression in individuals [2]. It has been shown that medications that increased serotonin in individuals elicit an increase in mood and affect [2].

With suicide rates in the United States (U.S.) continually rising and currently at an all-time high, providers are prescribing increasingly more selective serotonin reuptake inhibitors (SSRIs) to help combat depression [3-4]. Fluoxetine was synthesized in 1972 and, in 1987, was the first SSRI approved by the United States Food and Drug Administration for the treatment of major depression. Other uses for SSRIs include treatment for other psychiatric disorders such as panic disorder, obsessive-compulsive disorder, generalized anxiety disorder, social anxiety disorder, posttraumatic stress disorder, body dysmorphic disorder, bulimia nervosa, binge eating disorder, premenstrual dysphoric disorder, and somatoform disorders [5].

The widely prescribed nature of SSRIs has allowed for an increase in the incidence of drug dosage-related toxicity when careful monitoring does not take place. One notable dose-related toxicity known as serotonin syndrome is most often caused by the simultaneous ingestion of two or more serotonergic medications. Serotonin syndrome may be associated with the therapeutic error, idiopathic response, or intentional overdose [6]. Common culprits include SSRIs, monoamine oxidase inhibitors (MAOIs), tryptophan, triptans, cocaine, and more [7]. Serotonin syndrome presents in the clinical setting with a variety of neuromuscular, autonomic, and gastrointestinal symptoms due to the concentration of the serotonin receptors. Patients can present with symptoms such as heavy sweating, diarrhea, headache, muscle rigidity, confusion, rapid heart rate, high blood pressure, and agitation or restlessness [7]. Non-specific laboratory findings concurrent with SSRI use and serotonin syndrome include, but are not limited to, an elevated white blood cell count, elevated creatine phosphokinase, decreased serum bicarbonate concentration, and hyponatremia [5].

The diagnosis of serotonin syndrome remains a challenge because it is a clinical diagnosis and there is not one particular laboratory test that can be used for objective diagnosis. Three diagnostic criteria systems, the Sternbach (SC), Radomski (RC) and Hunter (HC) criteria, attempt to arrive at a diagnosis by utilizing a constellation of symptoms thought to indicate serotonin syndrome when presented together. SC and RC draw on neuromuscular, cognitive, and autonomic symptoms, while HC focuses on neuromuscular symptoms such as clonus, hyperreflexia, and tremor [8]. Although any of the three diagnostic criteria can be utilized, the Hunter Criteria is the most sensitive criteria for the diagnosis of serotonin syndrome [8]. Treatment of a patient with serotonin syndrome involves discontinuation of all drugs suspected to be involved and symptomatic management started promptly [7].

## Case presentation

A 64-year-old woman with a complex past medical history presented to the emergency department via ambulance after being found poorly responsive by her husband. Her husband stated that when he attempted to wake her up from a nap earlier that day, she was less responsive than usual and complained that she was unable to move her lower extremities due to stiffness. Upon questioning the patient, she admitted to shortness of breath and muscle stiffness but denied nausea, vomiting, diarrhea, headache, abdominal pain, chest pain, or a cough. She denied any recent falls or injuries.

The patient had a past medical history of anxiety, depression, hypertension, hyperlipidemia, neuropathy, fibromyalgia, stage 3 chronic kidney disease, chronic pain syndrome, and pseudotumor cerebri status post-ventricular atrial shunt. She denied any history of stroke or myocardial infarction. Home medications included 0.5 milligrams (mg) of clonazepam once daily, 100 mg lamotrigine once daily, 600 mg gabapentin twice daily, oxycodone 10 mg once daily pen, 20 mg citalopram twice daily, and paroxetine 25 mg controlled release in the morning and 12.5 mg at bedtime. The patient denied any alcohol, tobacco, or illicit drug use.

**Figure 1:**
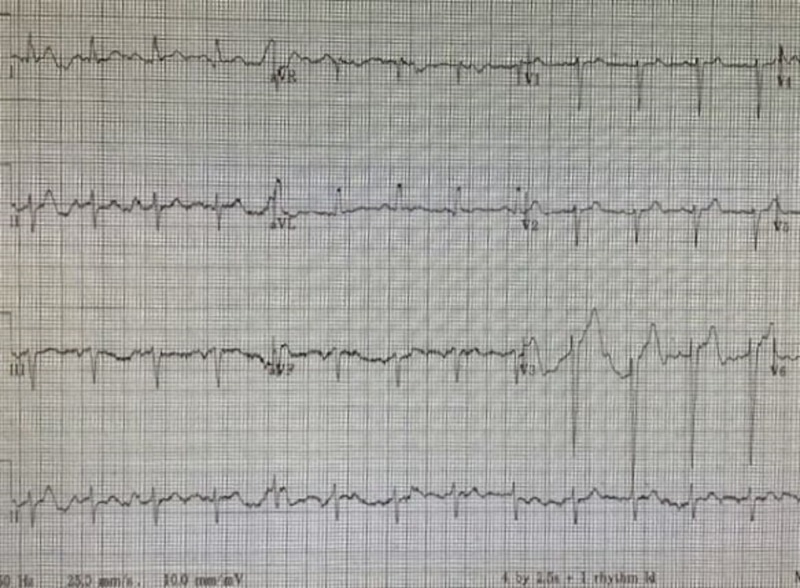
EKG of a Serotonin Syndrome Patient EKG: electrocardiogram

Upon presentation to the emergency department, the patient had a temperature of 101.1 ºF, heart rate of 107 beats per minute, 27 respirations per minute, and a blood pressure of 171/73 mmHg. A cardiovascular exam revealed regular rhythm with a systolic murmur grade III/VI, no edema, and palpable peripheral pulses. Lungs were clear to auscultation bilaterally, and the chest was non-tender. Abdominal exam revealed a soft, non-tender abdomen with normal bowel sounds and no masses. An electrocardiogram (EKG) revealed left ventricular hypertrophy, left anterior fascicular block, and sinus tachycardia with no other changes since previous EKG (Figure 1). Her lactic acid level was 5.0 mmol/L, and her influenza screen was negative. Her urinalysis was normal, and a chest radiograph revealed a right lower lobe infiltrate. The patient was then admitted to the hospital for a further work-up.

Until aspiration pneumonia could be ruled out, the patient was placed on a nothing-by-mouth (NPO) diet and was given intravenous (IV) antibiotics and fluids. Her home opioid medications were discontinued, with the exception of morphine as needed. A comprehensive neurological exam revealed disorientation and difficulties in attention, as well as hypertonia and hyperreflexia in the lower extremities with clonus bilaterally. A cranial computed tomography (CT) image showed an unchanged ventricular shunt and no acute process. Additionally, an electroencephalogram revealed generalized slowing and triphasic waves typically observed in neurodegenerative diseases or encephalopathy.

Despite adequate hydration, antibiotic use, and decreased administration of opioid medications, there were little improvements in her vitals or mental status. After a day in the hospital, her sodium level rose to 155 millimoles per liter and her creatine kinase level increased to 455 units per liter. She continued to have an alternating psychomotor agitation, lethargy, confusion, restlessness, stiffness, hyperreflexia, tachycardia, and uncontrolled hypertension.

The patient had been prescribed serotonergic and opioid medications for several years, but after the patient’s husband arrived, he was able to share a more extensive history and inform the care team that her psychiatrist had recently made significant changes to her drug regimen to address her increasing depression symptoms. According to the patient’s husband, a week prior to admission, the dosage of her SSRI, paroxetine, started to be tapered down, while the dosage of another SSRI, citalopram, was simultaneously being increased. Both medications were being prescribed concomitantly at high doses. Additionally, about one week prior to the hospital admission, her pain management specialist had discontinued her buprenorphine patch and started her on morphine. After learning about these recent medication changes, her care team discontinued her citalopram and paroxetine. Benzodiazepines were then prescribed for agitation.

Over the next 24-48 hours, the patient showed marked improvement in her mental status. The patient was noted to be more alert and oriented, conversing appropriately, with no agitation observed by her fifth day in the hospital. Her temperature, heart rate, blood pressure, and electrolytes simultaneously normalized. Education regarding the use of SSRIs, as well as other treatment options for depression and pain, were provided by the psychiatrist and medicine physician. After a few more days of monitoring, the patient was discharged to her home under her husband’s supervision with plans to begin psychotherapy, physical therapy, and occupational therapy.

## Discussion

This patient had a unique presentation of serotonin syndrome. At the peak of her symptoms, the patient experienced hypernatremia instead of the typical hyponatremia associated with SSRI medications [9]. SSRIs induce hyponatremia by enhancing the release of vasopressin, a hormone responsible for regulating the uptake/excretion of water from the kidneys [9]. Upon presentation to the emergency department, the patient had a normal sodium level of 135, but after a day in the hospital, it rose to 155. This atypical presentation of hypernatremia veered her providers from diagnosing serotonin syndrome sooner. Although serotonin syndrome typically presents with hyponatremia, the initiation of parental nutrition and nothing else by mouth may have induced a hypertonic dehydration.

Although associated with SSRIs in general, hyponatremia is not included in the Hunter Criteria for serotonin syndrome. As noted in Table 1, some signs and symptoms that are included in the Hunter Criteria are psychomotor agitation, hypertonia, inducible clonus, and a temperature above 100.4 ºF [7-8]. The patient presented with all of these, as well as with other indications of serotonin syndrome, such as elevations in blood pressure, heart rate, and creatine kinase levels [7]. Yet, the diagnosis of serotonin syndrome was not determined until several days into the patient’s hospital stay.

**Table 1:** Sternbach, Radomski, and Hunter Diagnostic Criteria Source: [8]

Sternbach	Radomski		Hunter
Coincidence with the addition or increase in a known serotonergic agent to an established treatment regimen, with at least three of the following features present:	Coincidence with the addition or increase in a known serotonergic agent (to an established treatment regimen) and the development of at least four minor or three major plus two minor symptoms:		In the presence of a serotonergic agent, the patient must also have a symptom or symptom constellation:
Mental status changes (confusion, hypermania) Agitation Myoclonus Hyperreflexia Diaphoresis Shivering Tremor Diarrhea Incoordination Fever	Major	Minor	Spontaneous clonus Inducible clonus AND agitation OR diaphoresis Ocular clonus AND agitation OR diaphoresis Tremor AND hyperreflexia Hypertonic AND temperature > 38 °C AND ocular clonus OR inducible clonus
	Mental		
	• Consciousness impairment • Elevated mood •Semicoma/coma	• Restlessness • Insomnia	
	Neurological		
	• Myoclonus • Tremor • Shivering • Rigidity • Hyperreflexia	• Uncoordination • Dilated pupils • Akathisia	
	Vegetative		
	• Fever • Sweating • Clinical features not an integral part of the underlying psychiatric disorder prior to commencing the serotonergic agent.	• Tachycardia • Tachy/dyspnea • Diarrhea •Hyper/hypotension	
Other etiologies (e.g. infectious, metabolic or endocrine, substance abuse or withdrawal) have been ruled out.	Other etiologies (e.g. infectious, metabolic or endocrine, substance abuse or withdrawal) have been ruled out.		
A neuroleptic drug had not been started or increased in dosage prior to the onset of the signs and symptoms listed above.	A neuroleptic drug had not been started or increased in dosage prior to the onset of the signs and symptoms listed above.		

This delayed diagnosis may be due to the difficulty in attaining a complete past medical history resulting from her altered mental status on initial presentation. She also had several complicating factors, including a possible aspiration pneumonia, which led providers in a different direction in her initial care. Fortunately, after much investigation and a more in-depth history from her husband, the diagnosis of serotonin syndrome was made, and her serotonergic medications withheld. After merely 24 hours of cessation of her serotonergic medications, the patient experienced a significant improvement in her mental status, normalized reflexes, and improvement of her electrolyte imbalances.

## Conclusions

Due to the increase of serotonergic prescriptions in the U.S., providers should have a heightened awareness of symptoms and a low threshold for diagnosing serotonergic toxicity. In our case, the diagnosis and treatment for serotonin syndrome were difficult due to multiple co-morbidities and an unreliable medication history. In addition, some of the providers were misled by the uncommon presentation of hyponatremia with serotonin syndrome. The initiation of parental nutrition and nothing else by mouth may or may not have induced a hypertonic dehydration resulting in hyponatremia. Regardless, the prolonged diagnosis could have been prevented if a thorough history and medication reconciliation was completed at intake. If more providers were familiar with the various diagnosis criteria, preferably the Hunter Criteria, due to its increased sensitivity, a prompter diagnosis could have taken place.
